# 

**Published:** 1895-10

**Authors:** 


### CONTENTS.

COMMUNICATIONS.

Dental Section of the American Medical Association Meeting at Baltimore, Md., May 6th, 1895 ..... 469
Gold as a Filling Material ..... 483
Generation and Degeneration of the Tissues of the Mouth ..... 491
Dental Law—Holland ..... 497
National Association of Dental Faculties ..... 500
National Association of Dental Examiners ..... 504

SELECTIONS.

Neuralgia of the Fifth Nerve—Treatment ..... 508
Nitroglycerine ..... 509
Double Dislocation of the Superior Maxilla ..... 510
Chemistry as a Servant of Anatomy ..... 511
Congress of Hygiene ..... 512
A New Medical Application of Electricity ..... 513
To Check Vomiting of Pregnancy ..... 513
Itching of the Mouth ..... 514
Ulyptol ..... 514
What Solder Can Do ..... 515
Some of the Causes of Neuralgia ..... 515
Properly Classed ..... 515

EDITORIAL.

Professional Standing ..... 516
A Law to Regulate the Practice of Medicine ..... 517
Removal ..... 519
To Examine Medical Students ..... 520
Dr. John S. Billings, U. S. Army ..... 520
The Final Volume of the Index Catalogue ..... 520

